# Frequency and prognostic implications of *KMT2A* rearrangements in children with precursor B-cell lymphoma

**DOI:** 10.1038/s41375-022-01757-0

**Published:** 2022-11-09

**Authors:** Rex K. H. Au-Yeung, Laura Arias Padilla, Martin Zimmermann, Sarah Reinke, Ilske Oschlies, Gabriele Escherich, Wilhelm Woessmann, Birgit Burkhardt, Wolfram Klapper

**Affiliations:** 1 Department of Pathology, Haematopathology Section and Lymph Node Registry, University of Kiel/University Hospital Schleswig-Holstein, Kiel, Germany; 2 Department of Pathology, the University of Hong Kong, Queen Mary Hospital, Hong Kong, Hong Kong; 3 Clinic of Pediatric Hematology and Oncology, University Hospital Münster, Münster, Germany; 4 Department of Pediatric Hematology and Oncology, Hannover Medical School, Hannover, Germany; 5 Clinic for Pediatric Hematology and Oncology, University Hospital Hamburg-Eppendorf, Hamburg, Germany

**Keywords:** B-cell lymphoma, Acute lymphocytic leukaemia, Translational research, Paediatrics, Cancer genetics

## To the Editor:

Precursor B-cell lymphoma (BCP-L) and leukemia (BCP-ALL), also known as B-lymphoblastic lymphoma/leukemia, are neoplasms of precursor cells committed to the B-cell lineage [1]. As agreed upon, BCP-L is distinguished from BCP-ALL by less than 25% bone marrow (BM) infiltration [1]. Many genetic subtypes of BCP-ALL were identified, some of which represent druggable targets, such as t(9;22) *BCR::ABL1* translocation (Philadelphia chromosome) [2]. *KMT2A* rearrangements are present in 2% of pediatric BCP-ALL > 1 year old and represent a poor prognostic factor, though recent studies showed that menin inhibitors are promising therapeutic options [3, 4]. Whereas the molecular genetic features of BCP-ALL have been extensively studied [5], data on BCP-L are sparse, and children and adolescents with BCP-L without evidence of leukemia are rare [6]. Children with BCP-L are stratified according to stage I/II vs. stage III/IV, but further strategies to stratify therapy are limited [7]. As *KMT2A* rearrangement and *BCR-ABL1* are poor prognostic factors but potentially druggable lesions in pediatric BCP-ALL, we analyzed the prevalence of these two genetic lesions in BCP-L and investigated whether they could serve as prognostic markers or therapeutic targets in these patients.

Patients between 1 to 18 years of age with BCP-L were identified in the files of the Department of Pathology, Hematopathology Section, Kiel, which is a reference pathology center for pediatric lymphomas in Germany for several decades. All patients with BCP-L had BM blast percentage < 25% assessed by BM aspiration smear. To avoid overestimation of BM blast percentage due to accidental puncture of osteolytic bone lesions, which is not uncommon in these patients [8], patients with focal bone lesions with >25% blasts but < 25% blast percentage in other BM puncture sites were also considered as BCP-L, as stated in clinical trial protocols [9]. Tissue microarrays (TMAs) were constructed from the formalin-fixed and paraffin-embedded (FFPE) tumour tissue. Fluorescence in-situ hybridization (FISH) and immunohistochemistry (IHC) were performed to screen for *KMT2A* breakpoints, fusion of *KMT2A* to known translocation partners, t(9;22) *BCR::ABL1* rearrangement, CD10, CD15 and NG2 expression. Clinical data were obtained from the German Pediatric Non-Hodgkin-Lymphoma Study Group (NHL-BFM) database. Patient sex, stage and sites of tumour involvement were analyzed with χ2 test, patient’s age distribution and BM blast percentage with Mann–Whitney U test, and mean LDH level with Student’s T-test. Kaplan-Meier curves of event-free survival (EFS) and overall survival (OS) were analyzed with log rank test. P-values < 0.05 were considered statistically significant. For bias analysis, the experimental cohort was compared to a control cohort in the NHL-BFM database, defined as patients with BCP-L but not included in the experimental cohort. Please refer to the supplement for details.

From 1996 to 2017, 61 patients with BCP-L between 1–18 years old were identified and had adequate tumour tissue for the retrospective analysis, including 48 cases of single lineage BCP-L, three cases of bi-lineage B/T-precursor cell lymphoma and ten cases of B/myeloid mixed phenotype lymphoma (Supplementary Fig. 1 and Supplementary Table 2). Two cases of bi-lineage B/T-precursor cell lymphoma was previously reported [10]. The immunophenotype of 17 cases of single lineage BCP-L were characterized in a previous study, but their genetic and clinical data have not been published before [11]. 58 out of 61 patients had adequate clinical data for bias analysis, and the experimental cohort contained a slightly higher proportion of patients less than ten years old (44/58 vs. 118/193, p = 0.043), though there was no significant difference in age distribution by Mann–Whitney U test. Otherwise, the experimental and control cohorts showed no significant difference in clinical parameters (supplementary table 1).

**Fig. 1:**
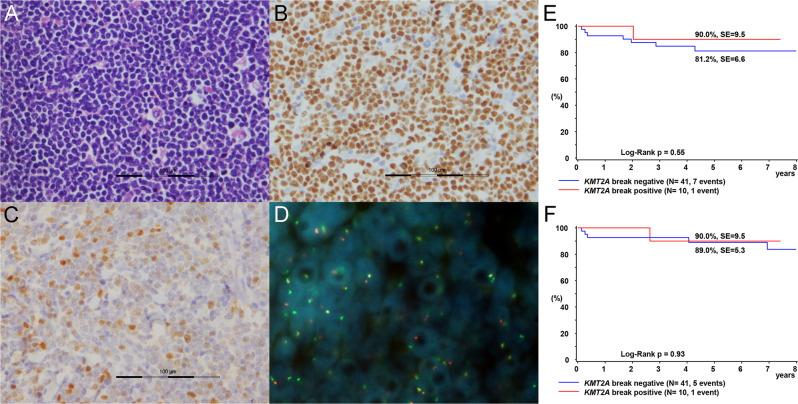
*KMT2A* break-positive precursor B-cell lymphoblastic lymphoma. **A**–**D** Histology of a representative case of BCP-L with positive *KMT2A* breakpoint (case KMT2A-BCPL-12). **A** Hematoxylin & eosin, (**B**) PAX5, (**C**). TdT and (**D**). fluorescence in-situ hybridization with *KMT2A* break-apart probe. **E** Five-year event-free survival and (**F**) overall survival of BCP-L patients according to *KMT2A*-break.

In the experimental cohort, 9/48 of BCP-L (19%), 2/3 of bi-lineage B/T-precursor cell lymphoma and 0/10 of B/myeloid mixed phenotype lymphoma were positive for *KMT2A* breakpoint (supplementary table 2). One case of bi-lineage B/T-precursor cell lymphoma had biallelic *KMT2A* breakpoints, which was reported in our previous study [10]. The frequency of *KMT2A* breakpoint-positive BCP-L in our cohort was much higher than the 2% prevalence of *KMT2A* rearrangements reported in non-infantile BCP-ALL [3]. A representative example of single lineage BCP-L with *KMT2A* breakpoint is shown in Fig. 1A–D. The clinical parameters and pathological features of the patients with *KMT2A*-positive BCP-L are described in supplementary tables 3 and 4.

Dual-fusion FISH analysis was performed successfully in 8/11 cases of *KMT2A*-positive BCP-L, including the bi-phenotypic cases. 1/8 case had t(6;11) *KMT2A::AFDN* fusion, 1/8 had t(9;11) *KMT2A::MLLT3* fusion and 2/8 had t(11;19) *KMT2A::MLLT1* fusion. The remaining 4/8 cases had no detectable fusion partner using the dual-fusion FISH probes, including t(4;11) *KMT2A::AFF1* (Supplementary Table 4). Due to limited biopsy material and suboptimal DNA quality in the archival FFPE tissue, we could not directly sequence the *KMT2A* breakpoint. No BCP-L with t(9;22) *BCR::ABL1* rearrangements was detected in our cohort (data not shown), in keeping with our previous study on lymphomas with multi-lineage differentiation [10].

On protein expression level, *KMT2A*-positive BCP-L (including bi-phenotypic cases) less often showed CD10 expression, compared to *KMT2A*-negative cases (5/11 vs. 43/49 cases, p = 0.005). The findings were consistent with BCP-ALL with *KMT2A* rearrangements [12]. There was no significant difference in CD15 expression between *KMT2A*-positive and negative cases (0/10 vs. 1/49, p = 1.0), contrasting findings reported in BCP-ALL [12]. Of note, *KMT2A* breaks were positively associated with mixed B/T phenotype and negatively associated with mixed B/myeloid phenotype (p = 0.033) (Supplementary table 2). This observation is consistent with our previous study, in which all cases with mixed B/myeloid phenotype were negative for *KMT2A* breakpoints [10]. There was also no significant difference in NG2 expression between *KMT2A* breakpoint-positive and negative BCP-L cases (4/11 vs. 18/45, p = 1.0, Supplementary Fig. 2).

The clinical features of our cohort are summarized in Table 1. One patient had trisomy 15, one patient had myotonic dystrophy, and one patient had ALL 11 years before the diagnosis of BCP-L. For this patient, there was no known genetic predisposition syndrome, the BCP-L was negative for *KMT2A* rearrangements, and the ALL and BCP-L showed different B-cell receptor gene rearrangement, although both tumors had t(12;21), hence the possibility of late relapse of t(12;21) ALL could not be entirely excluded. All other patients had no significant past history. Children with *KMT2A*-positive BCP-L were significantly younger than *KMT2A*-negative cases (median age 3.83 vs. 8.22 years, p = 0.047). Children with *KMT2A*-positive BCP-L more often showed skin involvement (6/11 vs. 4/45, p = 0.002). There was no significant difference in sex distribution, serum LDH level, CNS involvement, BM involvement, BM blast percentage and tumour stage between *KMT2A*-positive and negative cases (Table 1). Survival analysis was performed on a subset of 50 patients who received comparable treatment regimens according to NHL-BFM protocols without dose reduction. The five-year EFS and OS of patients with *KMT2A*-positive BCP-L were not significantly different to those patients without *KMT2A* break (Fig. 1E and F).

**Table 1:** Clinical and pathological features of patients with pediatric BCP-L with and without *KMT2A* breakpoint.

Features at disease presentation	*KMT2A* break- positive	*KMT2A* break- negative	P-value
Male sex	4/11 (36%)	25/47 (51%)	p = 0.505 (Fisher exact test)
Median age (range)	3.83 years (1.2-17.5)	8.22 years (2.0-17.8)	p = 0.047 (Mann–Whitney U test)
Stage (St. Jude/Murphy)			
Stage I	3/11 (27%)	3/47 (6%)	p = 0.373 (χ2 test)
Stage II	1/11 (9%)	8/47 (17%)	
Stage III	4/11 (36%)	16/47 (34%)	
Stage IV	3/11 (27%)	19/47 (40%)	
Stage B-ALL	0/11 (0%)	1/47 (2%)	
Mean serum LDH (Range)	288 U/L (265-320)	325 U/L (95-1118)	p = 0.659 (Student’s T-test)
Skin involvement	6/11 (55%)	4/45 (9%)	p = 0.002 (Fisher exact test)
CNS involvement	0/11 (0%)	6/47 (13%)	p = 0.583 (Fisher exact test)
Bone marrow involvement	4/11 (36%)	15/47 (32%)	p = 1.000 (Fisher exact test)
Median bone marrow blast percentage (range)	0% (0% - 13.0%)	0% (0% - 24.0%)	p = 0.941 (Mann–Whitney U test)
Positive CD10 expression	5/11 (46%)	43/49 (88%)	p = 0.005 (Fisher exact test)
Positive CD15 expression	0/10 (0%)	1/49 (2%)	p = 1.000 (Fisher exact test)
Positive NG2 expression	4/11 (36%)	18/45 (40%)	p = 1.000 (Fisher exact test)

To our knowledge, our study represented the largest cohort of BCP-L in the literature. As there was no significant bias detected in our cohort compared to other patients with BCP-L in the NHL-BFM database, it is reasonable to assume that our data are representative for pediatric BCP-L in central Europe. The prevalence of *KMT2A* rearrangements in our cohort is much higher than non-infantile BCP-ALL, and *KMT2A* rearrangements in BCP-L were not associated with unfavorable outcome, unlike BCP-ALL [3]. These findings suggest that, even with similar *KMT2A* rearrangements, BCP-L may have different biology compared to BCP-ALL and warrants further study.

Skin and subcutaneous involvement in pediatric BCP-L are common and affects 23% of patients [8]. Similar findings were also reported in adult BCP-L, and infantile BCP-ALL with *KMT2A* rearrangements [6, 13]. To our knowledge, except for isolated case reports [14], our study is the first to report that *KMT2A* break-positive BCP-L are significantly associated with skin involvement compared to *KMT2A*-negative cases, outside of infantile BCP-ALL cases.

Our analysis is limited regarding to the molecular features of BCP-L, since pediatric BCP-L is rare compared to BCP-ALL, and we were limited to archival BCP-L tissue that were small and preserved in FFPE blocks. Nevertheless, our data suggest that BCP-L is a valuable model for studying pathogenic mechanisms of BCP neoplasms, especially those with *KMT2A* aberrations. Detailed molecular genetic analysis will be required to understand which mechanisms lead to the clinical presentation as lymphoma instead of leukemia, and whether this clinical presentation is driven by genetic features of the tumor, or host dependent factors such as immunological status. These analyses will be technically challenging as biopsies of BCP-L are usually small and restricted to FFPE tissue, but they may answer the long-standing question of whether BCP-L and BCP-ALL are the same disease as defined in the WHO Classification of lymphoid neoplasm [1], or they are biologically distinct tumors.

In summary, 19% of BCP-L in our cohort were positive for *KMT2A* rearrangements, which is higher than BCP-ALL in this age group. No t(4;11) was detected. *KMT2A*-positive BCP-L is significantly associated with younger age and skin involvement, and negatively associated with myeloid phenotype. Unlike BCP-ALL, *KMT2A*-rearranged BCP-L is not associated with inferior outcome with standard NHL-BFM type therapy. Our data provide new understanding of the biology of this rare disease and shed light on future research directions on the biopsy of *KMT2A* and precursor lymphoid neoplasms, as well as the difference between BCP-L and BCP-ALL.

## Supplementary information

Supplementary material

## Data Availability

All data in this manuscript will be made available upon request.
