# Supplementary material for: Frequency and prognostic implications of KMT2A rearrangements in children with precursor B-cell lymphoma
Source: Leukemia. 2022 Nov 9;37(2):488–91. doi: 10.1038/s41375-022-01757-0 (PMC9898023; doi:10.1038/s41375-022-01757-0)
Supplement: Supplementary file 1 — Supplementary material [file 41375_2022_1757_MOESM1_ESM.docx]

**Supplementary data**

**Frequency and prognostic implications of *KMT2A* rearrangements in children with precursor B-cell lymphoma**

Rex K.H. Au-Yeung,^1,2^ Laura Arias Padilla,^3^ Martin Zimmermann,^4^ Sarah Reinke,^1^ Ilske Oschlies,^1^ Gabriele Escherich,^5^ Wilhelm Woessmann,^5^ Birgit Burkhardt,^3^ Wolfram Klapper^1^,*

^1^Department of Pathology, Haematopathology Section and Lymph Node Registry, University of Kiel/University Hospital Schleswig-Holstein, Kiel, Germany

^2^Department of Pathology, the University of Hong Kong, Queen Mary Hospital, Hong Kong

^3^Department of Pediatric Hematology and Oncology, University of Münster, Münster, Germany.

^4^Department of Pediatric Hematology and Oncology, Hannover Medical School, Hannover, Germany

^5^Clinic for Pediatric Hematology and Oncology, University Hospital Hamburg-Eppendorf, Hamburg, Germany.

*Corresponding author

**Material and methods**

Patient selection

To identify potential cases of BCP-L with *KMT2A* rearrangements, the pathology records and formalin-fixed and paraffin-embedded (FFPE) lymphoma tissue of all patients between 1-18 years of age with the diagnosis of BCP-L, bilineage B-/T-lymphoblastic lymphoma and B/myeloid mixed phenotype acute leukemia according to the WHO classification and retrieved from the Kiel Lymph Node Registry (years 1996-2017). Bone marrow blast percentage was assessed by bone marrow aspiration smears, and aspirates with blast percentage greater than 20% were assessed by flow cytometry. Patients with less than 5% tumour cells in the bone marrow were considered as BCP-L without bone marrow involvement, and patients with bone marrow blast percentage between 5% to 24% were considered as BCP-L with bone marrow involvement. To avoid overestimation of BM blast percentage due to accidental puncture of osteolytic bone lesions, which is not uncommon in these patients, patients with focal bone lesions with more than 25% blasts, but less than 25% blast percentage in other BM puncture sites, were also considered as BCP-L. All histological diagnoses were independently confirmed by national review pathologists (I.O. and W.K.). A flowchart describing the case selection process can be found in Supplementary figure 1.

Tissue microarray construction and fluorescence in-situ hybridization (FISH) studies

Tissue microarrays (TMAs) were constructed from the FFPE tumour blocks, composed of two cores of 0.6 mm diameter from each case. *KMT2A* break-apart fluorescence in-situ hybridization (FISH) was performed on the TMAs using commercially available probes (ZYTOVISION GmbH, Germany). Cases positive for *KMT2A* break-apart by FISH were screened for the translocation partners using fusion assays for t(4;11) *KMT2A::AFF1*, t(6:11) *KMT2A::AFDN*, t(9;11) *KMT2A::MLLT3* and t(11;19) *KMT2A::MLLT1* rearrangements (ZYTOVISION GmbH, Germany). The translocation t(9;22) *BCR::ABL1* was detected by a fusion assay (ZYTOVISION GmbH, Germany). The hybridization results were visualized on Zeiss Axioscope fluorescence microscope (Zeiss, Jena, Germany) and confirmed by two independent observers.

Immunohistochemical studies

Immunohistochemistry (IHC) for CD10 (clone 56C6, Becton Dickinson), CD15 (clone MMA, Leica Biosystems) and NG2 (rabbit polyclonal, Abcam) was performed retrospectively on the TMAs by automated IHC stainer (Leica BOND, Leica Biosystems, Germany) according to the manufacturer’s instructions as previously published.(1)

Clinical data and statistical analysis

Clinical data of each patient was retrieved from the German Pediatric Non-Hodgkin-Lymphoma Study Group (NHL-BFM) database, including sex, age, Murphy/St. Jude staging, serum lactate dehydrogenase (LDH) level at diagnosis, regional lymph node involvement, extranodal disease, central nervous system (CNS) or bone marrow involvement, bone marrow blast percentage, event-free survival and cumulative incidence of disease relapse. Statistical analysis was performed using IBM SPSS (IBM Corporation, Armonk, NY) and SAS-PC 9.4 software (SAS Institute, Cary, NC). Kaplan-Meier survival analysis was performed on the overall and event free survival data and compared using the log rank test. For each statistical test, two-tailed *p*-value of less than 0.05 was reported as statistically significant. Kaplan-Meyer Survival analysis by log-rank test was performed on patients who received comparable treatment regimens according to NHL-BFM protocols without dose reduction. Patients who were excluded from survival analysis included one patient who had dose-reduced therapy due to myotonic dystrophy, one patient who had ALL 11 years before the development of BCP-L, one patient who received other therapies before NHL-BFM protocols, one patient who changed to another therapy protocol after commencement of NHL-BFM protocols, three patients who received ALL-like therapy, and four patients with missing survival data.

Ethical approval

This research was approved by the Institutional Review Board / Ethics Committee of the medical faculty of the University of Kiel (review number D447/10) and conducted in accordance with the Declaration of Helsinki. All patients were registered in the clinical trials and/or registry of the BFM-NHL group and informed consent by the parents was obtained.

**References**

1. Oschlies I, Burkhardt B, Chassagne-Clement C, d'Amore ES, Hansson U, Hebeda K, et al. Diagnosis and immunophenotype of 188 pediatric lymphoblastic lymphomas treated within a randomized prospective trial: experiences and preliminary recommendations from the European childhood lymphoma pathology panel. *Am J Surg Pathol*. 2011;35(6):836-44.

**Supplementary Table 1. Bias analysis of the experimental cohort vs. control cohort.**

| **Clinical features** | **Experimental cohort** | **Control cohort** | **P-value** |
| --- | --- | --- | --- |
| **Male sex** | 29/58 (50%) | 101/193 (52%) | P=0.767  (Fisher exact test) |
| **Age <10 years** | 44/58 (76%) | 118/193 (61%) | **P=0.043**  (Fisher exact test) |
| **Median age**  **(range)** | 7.39  (1.2-17.8) | 8.45  (0.2-19.5) | P=0.234  (Mann-Whitney U test) |
| **Stage (St. Jude/Murphy)** |  |  |  |
| Stage I | 6/58 (10%) | 20/187 (11%) | P=0.380  (χ^2^ test) |
| Stage II | 9/58 (16%) | 43/187 (23%) |  |
| Stage III | 20/58 (35%) | 57/187 (31%) |  |
| Stage IV | 22/58 (38%) | 67/187 (36%) |  |
| Stage B-ALL | 1/58 (2%) | 0/187 (0%) |  |
| **Mean serum LDH (U/L)**  **(Range)** | 319  (95-1118) | 341  (99-1789) | P=0.757  (Student t test) |
| **CNS involvement** | 6/58 (10%) | 15/193 (8%) | P=0.589  (Fisher exact test) |
| **Bone marrow involvement** | 19/58 (33%) | 52/193 (27%) | P=0.408  (Fisher exact test) |
| **Median bone marrow blast percentage (range)** | 0%  (0% - 24%) | 0%  (0% - 23%) | P=0.205  (Mann-Whitney U test) |

**Supplementary Table 2. Prevalence of *KMT2A* breakpoint in pediatric patients with BCP-L.**

| **Tumor phenotype** | ***KMT2A* break-point positive** | ***KMT2A* break-point negative** | **P-value** |
| --- | --- | --- | --- |
| Precursor B-cell lymphoma | 9/48 (19%) | 39/48 (81%) | **p=0.030**  **(χ2 test)** |
| Bilineage B/T-precursor cell lymphoma | 2/3 (67%) | 1/3 (33%) |  |
| B/myeloid mixed phenotype lymphoma | 0/10 (0%) | 10/10 (20%) |  |
| Total | 11/61 (18%) | 50/61 (82%) |  |

**Supplementary Table 2. Clinical characteristics of the 12 patients with *KMT2A* breakpoint positive BCP-L.**

M=male; F=female; na=not available**.**

| **Case No.** | **Sex** | **Age in years** | **Tumour localization** | **Bone marrow**  **blast percentage** | **Stage**  **(St. Jude/**  **Murphy)** | **NHL-BFM**  **Risk group** | **Remarks** |
| --- | --- | --- | --- | --- | --- | --- | --- |
| KMT2A-BCPL-01 | F | 1.2 | Cervical lymph nodes, skin | 0% | III | Medium risk | Nil |
| KMT2A-BCPL-02 | F | 9.6 | Skin | 0% | I | Standard risk | Nil |
| KMT2A-BCPL-03 | F | 14.9 | Cervical and axillary lymph nodes, mediastinum, pericardial effusion, pleural effusion, bone marrow | 10% | IV | Medium risk | Nil |
| KMT2A-BCPL-04 | M | 4.1 | Abdominal and inguinal lymph nodes, intra-abdomen | 0% | III | Standard risk | Chemotherapy reduced due to myotonic dystrophy |
| KMT2A-BCPL-05 | M | 7.3 | Cervical lymph nodes | 0% | I | Standard risk | Nil |
| KMT2A-BCPL-06 | M | 17.8 | Cervical and axillary lymph nodes, upper aerodigestive tract, mediastinum and bone marrow | 7% | IV | Medium risk | Nil |
| KMT2A-BCPL-08 | M | 1.8 | Abdominal and inguinal lymph nodes, skin | 13% | IV | Medium risk | Nil |
| KMT2A-BCPL-09 | F | 3.7 | Ear | na | II | Standard risk | Nil |
| KMT2A-BCPL-10 | F | 3.8 | Skin and bone | 0% | III | Medium risk | Nil |
| KMT2A-BCPL-11 | F | 3.5 | Skin | 0% | III | Medium risk | Nil |
| KMT2A-BCPL-12 | F | 1.4 | Peripheral lymph nodes and soft tissue | 4%* | IV | Medium risk | Nil |

*Patient KMT2A-BCPL-12 had undifferentiated cells with FAB L1 morphology in two of the bone marrow smears, in addition to the 4% blast in the bone marrow. The undifferentiated cells and the blasts constitute 6-12% of the cells. Therefore, this patient was labelled as “positive for bone marrow involvement” for treatment purposes.

**Supplementary Table 3. Tumor typing, immunophenotype and molecular findings of the 12 patients with *KMT2A* breakpoint positive pediatric BCP-L**. BCP-L= precursor B-cell lymphoma, ++= strongly positive, += weakly positive, –= negative, na= not available.

| **Case No.** | **Tumor typing** | **CD19** | **CD20** | **PAX5** | **CD79a** | **CD3** | **CD10** | **CD15** | **MPO** | **TdT** | **NG2** | **Ki-67** | **Other IHC** | ***KMT2A* break-apart FISH** | ***KMT2A* fusion partner by FISH** | **t(9;22) *BCR::ABL1* fusion by FISH** |
| --- | --- | --- | --- | --- | --- | --- | --- | --- | --- | --- | --- | --- | --- | --- | --- | --- |
| KMT2A-BCPL-01 | BCP-L | na | – | na | ++ | – | – | – | na | + | – | 80% |  | Positive | Not detected | Negative |
| KMT2A-BCPL-02 | BCP-L | na | + | na | ++ | na | – | – | – | + | – | 50% | CD5– | Positive | Not detected | Negative |
| KMT2A-BCPL-03 | Bilineage B/T- precursor cell lymphoma | na | – | + | ++ | ++ | – | – | – | ++ | – | 80% | CD7++ | Positive  (biallelic break) | t(11;19) | Negative |
| KMT2A-BCPL-04 | BCP-L | na | – | na | ++ | – | ++ | na | na | ++ | – | na |  | Positive | Not detected | Negative |
| KMT2A-BCPL-05 | BCP-L | na | – | ++ | ++ | – | + | – | – | ++ | – | 50% |  | Positive | na | Negative |
| KMT2A-BCPL-06 | Bilineage B/T- precursor cell lymphoma | + | na | – | ++ | ++ | – | – | na | + | – | 70% | CD2+  CD5+ | Positive | t(6;11) | Negative |
| KMT2A-BCPL-08 | BCP-L | ++ | – | ++ | ++ | – | – | – | – | + | + | 80% |  | Positive | na | Negative |
| KMT2A-BCPL-09 | BCP-L | ++ | na | ++ | – | – | + | – | – | ++ | + | 80% |  | Positive | t(9;11) | Negative |
| KMT2A-BCPL-10 | BCP-L | ++ | – | na | + | – | ++ | – | – | ++ | – | 90% |  | Positive | na | Negative |
| KMT2A-BCPL-11 | BCP-L | ++ | + | ++ | ++ | – | ++ | – | – | ++ | + | 80% |  | Positive | t(11;19) | Negative |
| KMT2A-BCPL-12 | BCP-L | ++ | – | ++ | + | – | – | – | – | + | + | 50% |  | Positive | Not detected | Negative |

**Supplementary Figure 1.** **Flowchart of the case selection process.**

**

**

**Supplementary Figure 2. NG2 expression in a case of *KMT2A* breakpoint-positive pediatric BCP-L (KMT2A-BCPL-08).**

**
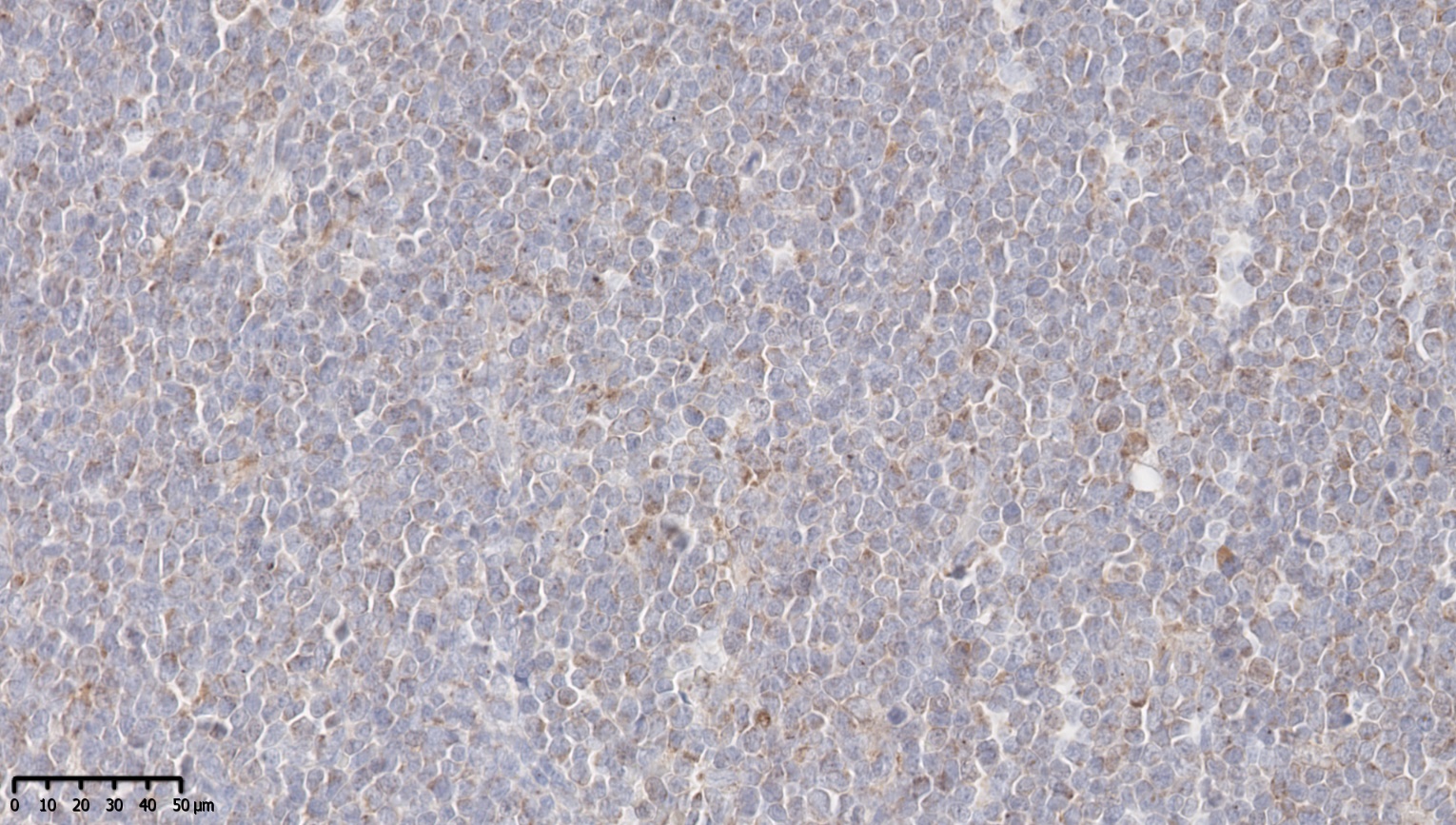
**
